# Use of the Magnetic Field for Improving Gyroscopes’ Biases Estimation

**DOI:** 10.3390/s17040832

**Published:** 2017-04-11

**Authors:** Estefania Munoz Diaz, Fabian de Ponte Müller, Juan Jesús García Domínguez

**Affiliations:** 1 German Aerospace Center (DLR), Institute of Communications and Navigation, Oberpfaffenhofen, 82234 Wessling, Germany; fabian.pontemueller@dlr.de; 2 Electronic Department from the University of Alcalá, E.P.S. Campus Universitario, s/n, 28806 Alcalá de Henares, Spain; jjesus.garcia@uah.es

**Keywords:** orientation, pedestrian, perturbed magnetic field, homogeneous magnetic field, inertial navigation

## Abstract

An accurate orientation is crucial to a satisfactory position in pedestrian navigation. The orientation estimation, however, is greatly affected by errors like the biases of gyroscopes. In order to minimize the error in the orientation, the biases of gyroscopes must be estimated and subtracted. In the state of the art it has been proposed, but not proved, that the estimation of the biases can be accomplished using magnetic field measurements. The objective of this work is to evaluate the effectiveness of using magnetic field measurements to estimate the biases of medium-cost micro-electromechanical sensors (MEMS) gyroscopes. We carry out the evaluation with experiments that cover both, quasi-error-free turn rate and magnetic measurements and medium-cost MEMS turn rate and magnetic measurements. The impact of different homogeneous magnetic field distributions and magnetically perturbed environments is analyzed. Additionally, the effect of the successful biases subtraction on the orientation and the estimated trajectory is detailed. Our results show that the use of magnetic field measurements is beneficial to the correct biases estimation. Further, we show that different magnetic field distributions affect differently the biases estimation process. Moreover, the biases are likewise correctly estimated under perturbed magnetic fields. However, for indoor and urban scenarios the biases estimation process is very slow.

## 1. Introduction

In Europe and North America, people spend more than 90% of their time indoors [1], which is a satellite-denied environment. The indoor scenario is the base of highly demanded mass market applications like guidance in shopping malls or professional applications for rescue personnel, among others. The navigation of pedestrians based on inertial measurement units has experienced great growth in recent years due to the miniaturization and the price reduction of the micro-electromechanical sensors (MEMS). These sensors are nowadays embedded in every smartphone, that is usually carried in the front pocket of the trousers. Thus, we use pocket-mounted inertial sensors. On the one hand, MEMS inertial sensors are plentiful and inexpensive. On the other hand, their noise characteristics cause ever growing errors when processing their measurements.

One of the critical tasks of pedestrian navigation is the orientation estimation of MEMS inertial sensors due to their noise characteristics. The orientation of the pedestrian and the orientation of the sensor are tightly coupled if the sensor is attached to the pedestrian’s body. The estimation of the orientation for inertial pedestrian navigation, specially the estimation of the heading angle, has been widely described and analyzed in the state of the art [2,3,4,5,6,7]. Acceleration and turn rate measurements are used to estimate the orientation. Additionally magnetic field measurements allow enhancing the orientation estimation [8].

Magnetometers, which are commonly embedded together with the inertial sensors, measure magnetic fields, e.g., the Earth’s magnetic field. For centuries, Earth’s magnetic field has been used for determining the direction of the magnetic North. The angle between the magnetic and the geographic North is called declination. Facing the magnetic North, the angle the field makes with the horizon is called inclination. The values of intensity, inclination and declination change with time for the same location and are different for different locations on the Earth. Taking the advantage that the direction and magnitude of the field are known, by measuring only the Earth magnetic field it is possible to partially determine the orientation of the sensor. The complete orientation can not be computed because the angle representing the rotation around the axis parallel to the magnetic field is not determined.

The magnetometer has historically been discarded for indoor navigation applications, since the Earth’s magnetic field is usually perturbed due to the proximity of ferromagnetic materials and electric currents [6,9]. The magnetic North can only be clearly determined under homogeneous magnetic fields, which are difficult to find in indoor environments. However, an alternative way to extract valuable information for the orientation estimation out of a perturbed magnetic environment has recently been proposed. This procedure makes use of the fact that the aforementioned distortion is highly modulated in space, but relatively constant in time [10].

Along with the orientation angles, it is convenient to estimate also the biases of the gyroscopes [11,12,13,14,15]. Biases are systematic errors that have to be subtracted from the raw turn rate measurements. The biases for MEMS gyroscopes affect severely the orientation estimation if they are not compensated before integrating the turn rate measurements. During a walk under a magnetically contaminated environment, there are periods of constant or quasi-constant magnetic field. Although magnetic measurements corresponding to these periods are perturbed, they are all equally perturbed, thus the change in orientation of the magnetic vector is only due to the change in orientation of the pedestrian. The idea of using magnetic field measurements to estimate the biases of the gyroscopes was proposed in [6] for a sensor held in the hand. A basic analysis of its performance can be found in [16]. In the article, the estimation of the biases of the gyroscopes is analyzed with real measurements. However, the biases are not known in advance, therefore it is difficult to assess if the estimation converges to the correct value. The article concludes with the clear statement that the proposed correction does not significantly decrease the estimated gyroscopes error state variance. This correction is only helpful if low-cost gyroscopes and magnetometers are used [16].

A similar proposal for estimating the biases of the gyroscopes using magnetic field measurements was also suggested in [17] for a foot-mounted sensor. The analysis of its performance for the yaw angle is evaluated using synthetic signals. The experiment reveals that processing the synthetic signals with an unscented Kalman filter, the proposed correction decreases the error in the yaw angle after 100 s. However, neither the estimation of the biases of the gyroscopes, nor the magnetometer calibration in order to correctly apply the correction are covered. Additionally, the article claims that the correction can only be applied during the stance phase of the foot. It has been demonstrated, that the correction requires a constant or quasi-constant magnetic field [6], which is possible in wider periods rather than only during the stance phases of the foot.

Therefore, the idea of using magnetic field measurements during quasi-constant periods in order to estimate the biases of the gyroscopes has been proposed in the state of the art. However, the effectiveness of this method has not been properly proved yet. The aim of this work is to evaluate the use of magnetic field measurements in the process of estimating the gyroscopes biases for medium-cost MEMS sensors and the effectiveness of this correction. For our evaluation we have recorded a walk using a fiber optic gyroscopes to have error-free turn rate measurements to which we add a known bias. Then we have emulated a variety of magnetic fields, homogeneous and perturbed, in different locations on the Earth that allow replicating the same conditions of the experiment and only changing the magnetic field to analyze its influence on the biases estimation process. Additionally, the effect of the biases estimation on the yaw angle is evaluated. Finally, a long walk recorded with medium-cost MEMS magnetometers and gyroscopes is also analyzed in order to endorse the previous results obtained with emulated magnetic measurements and error-free turn rate measurements. The effect on the estimated trajectory of using magnetic field measurements to estimate the biases of the gyroscopes, as proposed in the state of the art, is studied using the previously mentioned walk.

Section 2 details the methods used for the proposed evaluation: first the orientation estimation algorithm used in this work and last the emulation of different magnetic field distributions. The results, which are summarized in Section 3, are clustered in two groups: first the evaluation of the error-free turn rate measurements with added known bias and emulated magnetic fields, and last the evaluation of the measurements recorded with medium-cost MEMS sensors. Finally, the conclusions of this work are drawn in Section 4.

## 2. Methods Used for the Evaluation

In this section, we describe the methods used for the proposed evaluation. First we detail the orientation estimation algorithm, which consists of a Kalman filter. Lastly, the procedure to emulate different magnetic field distributions from error-free turn rate measurements is described. In this subsection, we also detail how we add noise and biases to the error-free measurements in order to obtain similar quality measurements as if they were recorded with MEMS sensors.

### 2.1. Orientation Estimation Filter

The orientation estimation algorithm aims at fusing the information of gyroscopes, accelerometers and magnetometers in an optimal way to obtain the orientation of the sensor [18]. The Kalman filter is suited for modelling continuous variables whose system model equations are linear and whose system and measurements noise are Gaussian [19]. Taking into account the non-linearities of the equations involved in the orientation computation, it is convenient to use an extended Kalman filter [6] or an unscented Kalman filter [4,17].

The state vector of the filter proposed in this work, $x^{k}$, is composed by the Euler angles and the biases of the gyroscopes

(1) $$x^{k}={\left[\phi^{k},\theta^{k},\psi^{k},b_{x}^{k},b_{y}^{k},b_{z}^{k}\right]}^{T},$$

being ϕ the roll angle, θ the pitch angle, ψ the yaw angle, which are the Euler angles that represent the orientation, and $b_{i}$ the biases of the gyroscopes, for i={x,y,z} representing the three orthogonal axes. Although the error state Kalman filter is more common in navigation applications, we have chosen the full state Kalman filter because both have similar performance, as demonstrated in [20].

**Figure 1:**
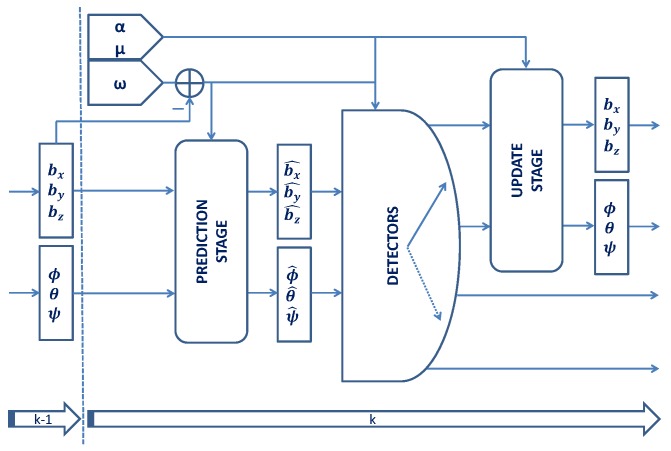
Diagram of the Kalman filter approach used in this work, where α, μ and ω represent the accelerometers, magnetometers and gyroscopes measurements respectively.

The diagram (Figure 1) shows the computation taken for the time stamp *k*. The variables α, μ and ω represent the measurements of the accelerometers, magnetometers and gyroscopes respectively. The states $\psi_{0}$ as well as $b_{0}$ are initialized to zero. The states $\phi_{0}$ and $\theta_{0}$, as well as the initial value of $\alpha_{0}$, $\mu_{0}$ and $\omega_{0}$ are computed during the calibration phase taking into account the average of 1 s of static measurements. After the prediction stage, the state vector is represented as ${\hat{x}}^{k}$. Next, some characteristics of the measurements are checked by the detectors, whose goal is to determine whether the updates can be applied or not. If the update stage takes place, the state vector is updated, $x^{k}$, and the $k^{th}$ iteration finishes. If no update takes place, the predicted state vector ${\hat{x}}^{k}$ is outputted and the $k^{th}$ iteration finishes.

#### 2.1.1. Prediction Stage

**Figure 2:**
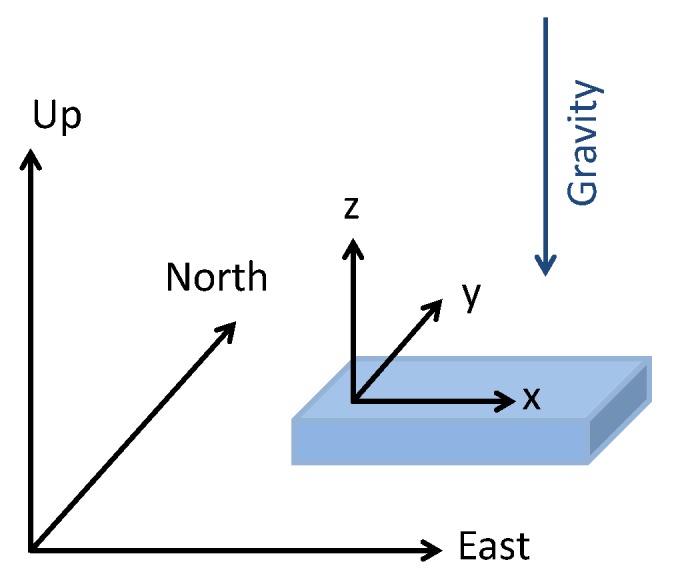
East-North-Up navigation frame and sensor frame representation.

The navigation frame we use in this work is the East-North-Up coordinate system (see Figure 2). The gyroscopes measure in body frame the turn rate of the sensor with respect to the inertial frame, $\omega_{\mathrm{ib}}^{b}$. The outputted turn rate is composed of:

(2) $$\omega_{\mathrm{ib}}^{b}=\omega_{\mathrm{nb}}^{b}+\underset{\omega_{\mathrm{in}}^{b}}{\underset{︸}{\omega_{\mathrm{en}}^{b}+\omega_{\mathrm{ie}}^{b}}}.$$

The term $\omega_{\mathrm{nb}}^{b}$ is the turn rate of the sensor measured in body frame with respect to the navigation frame. The term $\omega_{\mathrm{en}}^{b}$ is the transport rate, which represents the turn rate of the navigation frame with respect to the Earth-fixed frame. The term $\omega_{\mathrm{ie}}^{b}$ represents the turn rate of the Earth with respect to the inertial frame. Lastly, the term $\left(\omega_{\mathrm{en}}^{b}+\omega_{\mathrm{ie}}^{b}\right)$ is equivalent to $\omega_{\mathrm{in}}^{b}$, which represents the rotation of the navigation frame with respect to the inertial frame.

The turn rate of the sensor is given by the term $\omega_{\mathrm{nb}}^{b}$. However, the gyroscopes measure $\omega_{\mathrm{ib}}^{b}$, thus the term $\omega_{\mathrm{in}}^{b}$ must be compensated. Particularly for pedestrian navigation, the transport rate is negligible, because the travelled distances are not large enough to significantly change its latitude and longitude within a time stamp. The Earth rotation, which is approximately 15 ${}^{\circ }\;h^{-1}$, is usually not compensated because the noise of the MEMS gyroscopes is much greater. Therefore, it is assumed that $\omega_{\mathrm{nb}}^{b}\approx \omega_{\mathrm{ib}}^{b}$.

In this work, for practical reasons, we will use ω to shorten the term $\omega_{\mathrm{ib}}^{b}$. The estimation of the Euler angles is derived from the angular rate at each time stamp $\omega^{k}={\left[\omega_{x}^{k},\omega_{y}^{k},\omega_{z}^{k}\right]}^{T}$.

To compute the Euler angles (see [21]), first the biases have to be subtracted from the angular rate.

(3) $$\omega^{k}=\omega^{k}-b^{k-1}.$$

Then, the corrected turn rate measurements are integrated to compute the orientation. The orientation can be represented as a direct cosine matrix, C, which is a 3×3 rotation matrix in which each column is a unit vector along the sensor axes specified in terms of the navigation axes. The rotation matrix, the Euler angles and the quaternions are analogue ways of representing the orientation. In this work we use Euler angles instead of quaternions because the pitch angle never reaches 90 ${}^{\circ }$ while walking. For better understanding of the range values of the pitch angle, see [22].

The orientation at the time stamp *k* is the orientation at the time stamp k−1 rotated by the change in orientation that took place within the last δt seconds, represented in a matrix form as $A^{k}$:

(4) $${\hat{C}}^{k}=C^{k-1}\;\cdot \;A^{k},$$

being $A^{k}$

(5) $$A^{k}=I+\frac{\sin(\sigma )}{\sigma }\;\cdot \;B^{k}+\frac{1-\cos(\sigma )}{\sigma^{2}}\;\cdot \;{B^{k}}^{2},$$

where $\sigma =|\omega^{k}\delta t|$ and

(6) $$B^{k}=\left(\begin{array}{ccc} 0 & -\omega_{z}^{k}\delta t & \omega_{y}^{k}\delta t\\ \omega_{z}^{k}\delta t & 0 & -\omega_{x}^{k}\delta t\\ -\omega_{y}^{k}\delta t & \omega_{x}^{k}\delta t & 0\; \end{array}\right).$$

Therefore, the predicted Euler angles are extracted from

(7) $${\hat{C}}^{k}\equiv {\left[{\hat{\phi }}^{k},{\hat{\theta }}^{k},{\hat{\psi }}^{k}\right]}^{T}.$$

As Equation (1) shows, the biases of the gyroscopes are included in the state vector together with the Euler angles. In order to present the prediction model of the biases, we first introduce our error model of the gyroscopes. The turn rate measurements, $\omega^{k}$, can be represented as

(8) $$\omega^{k}={\tilde{\omega }}^{k}+e^{k},$$

being ${\tilde{\omega }}^{k}$ the error free turn rate and $e^{k}$ the measurement error. The turn rate error can be decomposed in two errors

(9) $$e^{k}=b^{k}+v^{k},$$

where $b^{k}$ is the bias systematic error and $v^{k}$ is the sensor noise that can be modelled as Gaussian white noise. To determine the biases error we choose an auto-regressive model of order one (AR1) [23]. The AR1 model is defined as

(10) $${\hat{b}}^{k}=c\;\cdot \;b^{k-1}+n^{k}.$$

The biases follow an exponentially correlated noise term defined in the AR1 model as the constant *c*, which is equal to the exponent $e^{-\frac{1}{\tau }}$, where τ is the correlation coefficient and $n^{k}$ can be modelled as Gaussian white noise with standard deviation $\sigma_{n}$. Thus, the predicted biases are

(11) $${\hat{b}}^{k}={\left[{\hat{b}}_{x}^{k},\hat{b_{y}^{k}},{\hat{b}}_{z}^{k}\right]}^{T}.$$

Therefore, Equation (7) and Equation (11) represent the vector state after the prediction stage. The values of the variance-covariance matrix of the state vector x stem from the noise of the gyroscopes through matrix $A^{k}$ for the Euler angles and from the model presented in Equation (10) for the biases.

#### 2.1.2. Detectors

We want to use the accelerometer and the magnetometer to extract additional orientation information, however, these sensors should not be used continuously. We have implemented two detectors that allow using the accelerometer and the magnetometer measurements only within the right periods.

While for foot-mounted sensors the zero velocity update (ZUPT) is used [24,25], for pocket-mounted sensors these periods of zero velocity do not exist, thus we use the acceleration. An accelerometer is capable of measuring specific force, whose units are $m\;s^{-2}$. The movement of the sensor provokes accelerations that are measured as well as the gravity. In order to properly use the acceleration measurements for the orientation estimation, only the gravity acceleration has to be measured. The *zero acceleration detector* identifies during the walk the periods of zero or quasi-zero acceleration due to specific movements of the sensor. Taking the advantage that the direction and magnitude of the field are known, by measuring only the gravity, it is possible to determine the roll and pitch angles of the sensor. The yaw angle cannot be determined, since it describes rotations around the axis parallel to the gravity vector [26].

The *magnetic disturbances detector* identifies during the walk the periods of constant or quasi-constant magnetic field. This is necessary because the measured magnetic field is often perturbed in indoor environments and the proposal of the state of the art we want to evaluate in this work can only be applied within these periods [26].

#### 2.1.3. Update Stage

There are different updates that can be applied to enhance the orientation obtained from the turn rate measurements. The authors in [27] propose an update using the gravity field. There is also an update based on magnetic field measurements applied during constant or quasi-constant periods, which has been proposed in [6] and further analyzed in [16]. It is also possible to use the homogeneous magnetic field to compute the yaw angle of the sensor, but indoor and urban scenarios rarely present homogeneous magnetic fields. Turn rate measurements can also be used under the assumption that no rotation is undergoing, therefore any measured turn rate is due to gyroscopes biases. This update has been proposed in [28]. Two updates based on acceleration and magnetic field measurements will be analyzed in detail in this work: the *Absolute Gravity Update* and the *Differential Magnetic Field Update*.

##### Absolute Gravity Update

The knowledge of the gravity field yields an estimation of the attitude if the acceleration due to the movement of the sensor is zero or quasi zero, then ∥α∥≃ 9.8 $m\;s^{-2}$. We use the *zero acceleration detector* to identify these periods.

Within the periods that the aforementioned condition is fulfilled, the attitude angles roll and pitch can be extracted as follows:

(12) $$\bar{\phi }=\arctan\left(\frac{\alpha_{y}}{\alpha_{z}}\right)$$

and

(13) $$\bar{\theta }=\arctan\left(\frac{-\alpha_{x}}{\sqrt{\alpha_{y}^{2}+\alpha_{z}^{2}}}\right),$$

where $\bar{\phi }$ and $\bar{\theta }$ represent the Euler angles roll and pitch and $\alpha_{i}$ for i={x,y,z} represents the acceleration reading for the *i*-axis measured in the sensor frame.

Then, the update equation yields:

(14) $$x^{k}={\hat{x}}^{k}+K_{a}\;\cdot \;\left({\left[\bar{\phi },\bar{\theta }\right]}^{T}-{\left[\hat{\phi },\hat{\theta }\right]}^{T}\right),$$

where $K_{a}$ is the Kalman gain [19]. The variance-covariance matrix of $\bar{\phi }$ and $\bar{\theta }$ is equal to the mutually uncorrelated noise of the accelerometers transformed by Equations (12) and (13).

##### Differential Magnetic Field Update

This update requires a constant or quasi-constant magnetic field. We use the *magnetic disturbances detector* to identify these periods.

The magnetic field at the current time stamp can be computed applying the rotation of the last δt seconds, $A^{k}$, to the magnetic field measured at the previous time stamp, $\mu^{k-1}$, as follows:

(15) $${\bar{\mu }}^{k}=A^{k}\;\cdot \;\mu^{k-1}.$$

Then, the update equation yields:

(16) $$x^{k}={\hat{x}}^{k}+K_{m}\;\cdot \;\left(\mu^{k}-{\bar{\mu }}^{k}\right),$$

being $\mu^{k}={\left[\mu_{x}^{k},\mu_{y}^{k},\mu_{z}^{k}\right]}^{T}$ the measured magnetic field and $K_{m}$ the Kalman gain. The variance-covariance matrix of $\bar{\mu }$ incorporates the mutually uncorrelated noise of the magnetometers and the variance-covariance matrix of $A^{k}$, which contains the noise of the gyroscopes.

The *Differential Magnetic Field Update* has been proposed in the state of the art in [6,17] to estimate the biases of the gyroscopes. The estimation of the biases is modified by this update because the biases are directly included in the update Equation (15) through the A matrix (see Equations (4) and (5)). The effectiveness of this update will be evaluated in this work.

### 2.2. Generation of the Emulated Magnetic Field

In this section, we detail the computation needed to emulate magnetic field measurements using error-free turn rate measurements. Additionally we define the noises we add to the error-free measurements in order to obtain similar quality measurements as if they were recorded with MEMS sensors. Lastly, we validate the fiber optic gyroscopes (FOG) turn rate measurements as quasi-error-free.

**Figure 3:**
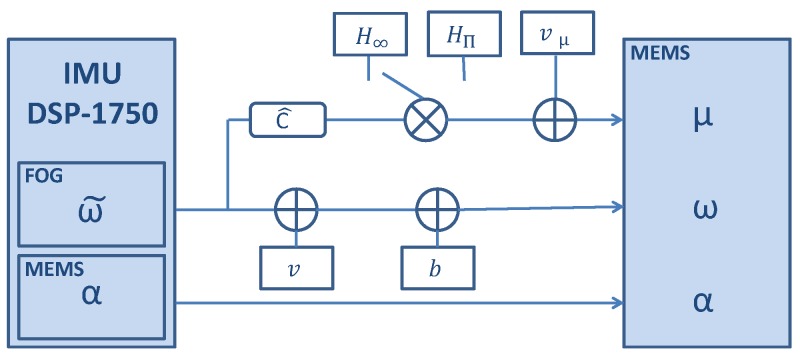
Diagram of the IMU DSP-1750 which includes the post-processing to emulate the magnetic measurements and the addition of noise to the turn rate measurements.

We will use the Inertial Measurement Unit (IMU) DSP-1750 from KVH [29], which embeds a FOG and a MEMS accelerometer (see Figure 3). The acceleration measurements, α, contain noise and biases characteristic from the MEMS. The turn rate measurements of the FOG, $\tilde{\omega }$, are considered error-free. These error-free turn rate measurements are used to emulate different magnetic field measurements, μ, as indicated in the diagram of Figure 3.

In order to emulate MEMS gyroscopes measurements ω we artificially add white Gaussian noise v and biases b to the FOG measurements as follows:

(17) $$\omega =\tilde{\omega }+v+b.$$

The noise and biases values have been chosen based on the typical values of the MEMS MTw from Xsens [30] and we will detail them in the evaluation section.

To emulate magnetic measurements μ the orientation of the sensor $\hat{C}$ is computed from the error-free turn rate measurements and multiplied by the magnetic field H. Finally white Gaussian noise $v_{\mu }$ is added as follows:

(18) $$\mu ={\hat{C}}^{T}\;\cdot \;H+v_{\mu },$$

where H is either the homogeneous magnetic field $H_{\infty }$ or the perturbed magnetic field $H_{\pi }$. The $v_{\mu }$ value is chosen based on the noise of the magnetometer integrated in the medium-cost MEMS MTw from Xsens and we will detail it in the evaluation section.

The election of the chosen locations on the Earth is due to their different magnetic field distributions. The homogeneous magnetic field values, $H_{\infty }$, have been rounded compared to the real values: for example, the Munich Earth Observatory has reported an average intensity of 48 μT, an inclination angle of 64.23 ${}^{\circ }$ and a declination angle of 2.57 ${}^{\circ }$ for April 2015 [31]. Table 1 shows all the homogeneous magnetic fields, $H_{\infty }$, expressed in East-North-Up coordinates and measured in μT.

In order to generate the perturbed magnetic field, $H_{\pi }$, we compute analytically the equations of a homogeneous field perturbed by one single ferromagnetic object. Then, we create a template of several objects. The ferromagnetic object is an iron cylinder with infinite length along the Up-axis with relative permeability $\mu_{\text{r}}=200$. Due to this geometry, the infinite cylinder will not influence the resulting perturbed magnetic field in the Up-axis. We generate a perturbed magnetic field from the homogeneous magnetic field of the Equator (Table 1). The inner magnetic field of the object is not considered, since the pedestrian cannot step into the cylinder.

The magnetic field can be derived from the Maxwell’s equations, which are four partial differential equations that describe how electric and magnetic fields are generated an altered by each other and by charges and currents. If the magnetic field is static, the time derivatives are zero and the system of equations decouples. The magnetic strength vector field can be expressed as the gradient of a scalar potential field, Ψ:

(19) H=−∇Ψ.

Since the influence of the ferromagnetic object on the homogeneous magnetic field decays with increasing distance, the solution of the boundary value, −∇Ψ, at infinite distance converges to the homogeneous magnetic field $H_{\infty }$, in this case the Equator.

(20) $$\lim_{r\to \infty }-\nabla \Psi =H_{\infty }.$$

The desired magnetic field must fulfill the boundary condition of Equation (19), therefore the solution yields [32]

(21) $$\begin{array}{c} -\nabla \Psi =\left|\right|H_{\infty }\left|\right|\;\cdot \;\frac{\mu_{r}-1}{\mu_{r}+1}\;\cdot \;\frac{r_{c}}{r}\;\cdot \;\sin\left(2\rho \right)\;\cdot \;u_{E}+\\ \left|\right|H_{\infty }\left|\right|\;\cdot \;\left(1+\frac{\mu_{r}-1}{\mu_{r}+1}\;\cdot \;\frac{r_{c}}{r}\;\cdot \;\cos\left(2\rho \right)\right)\;\cdot \;u_{N}, \end{array}$$

being $u_{E},u_{N}$ the unity vectors in East and North direction respectively, $r_{c}$ the radius of the cylinder, $\mu_{r}$ the relative permeability of the material of the cylinder,

(22) $$r=\sqrt{x^{2}+y^{2}}$$

and

(23) $$\rho =\arctan\left(\frac{x}{y}\right).$$

**Figure 4:**
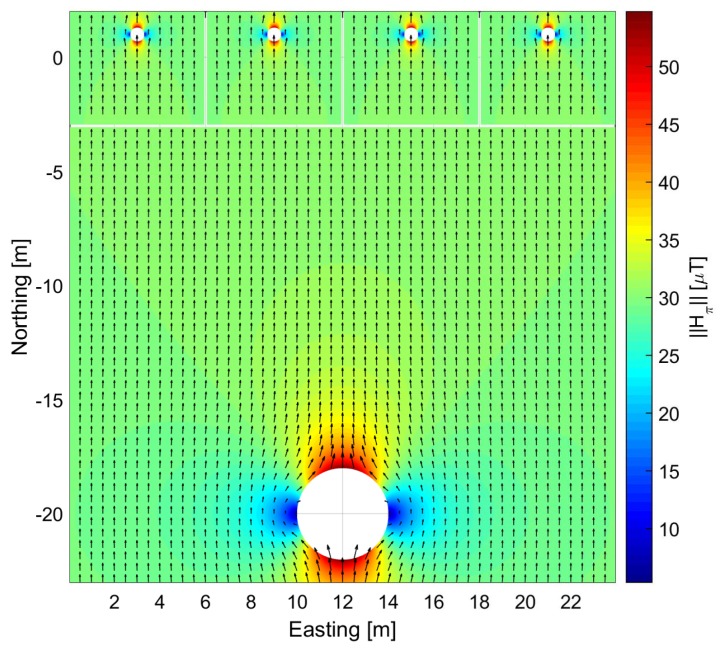
Generated template for a perturbed magnetic field $H_{\pi }$ including four streetlights and a parked car. The color codes the magnetic field intensity, measured in μT, and the arrows represent the orientation.

Using Equation (21) and the reference magnetic field of the Equator, two different fields have been generated: one perturbed by a cylinder of $r_{c}=30\;cm$ and another perturbed by a cylinder of $r_{c}=2\;m$. We have then composed a magnetic field template with four small cylinders representing streetlights and one big cylinder representing a parked car (Figure 4). As previously mentioned, we do not consider the influence of all cylinders simultaneously. In order to ensure that the *Differential Magnetic Field Update* is correctly applied, we will not apply the update if the pedestrian walks over the transition of two fields.

#### FOG Turn Rate Measurements

**Figure 5:**
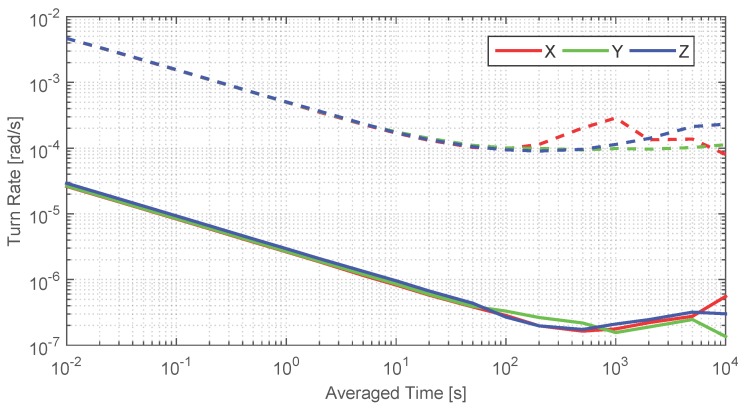
Allan deviation analysis of the FOG and the gyroscopes of the MEMS MTw in continuous and dashed line, respectively.

To assess that the FOG measurements can be considered quasi-error-free, 14 h of static turn rate measurements have been recorded to compute the Allan deviation shown in Figure 5. The continuous lines depict the noise analysis of the FOG and the dashed lines depict the noise analysis of the gyroscopes of the MEMS MTw, see [33]. The colors represent the mutually orthogonal gyroscopes in the axes *x*, *y* and *z*.

The noise analysis of both sensors shows a decreasing trend on the left side of the plot, where the white noise is dominant. This value can be directly extracted by intersecting the curves at 1 s. The FOG white noise is two orders of magnitude lower than the medium-cost MEMS gyroscopes white noise. On the right side of the plot, the Allan deviation shows a change in the trend. In this region the biases, i.e., the slow changing errors, become dominant. The bias stability, *B*, is located at the minimum. The standard deviation of the biases noise is

(24) $$\sigma_{n}=\frac{B}{\sqrt{\tau }},$$

being τ

(25) $$\tau =t_{B}\;\cdot \;f_{s},$$

where $t_{B}$ is the averaged time corresponding to the *B* and $f_{s}$ is the sampling frequency. Therefore, the lower the *B* value and the greater the $t_{B}$ are, the more stable are the biases of the gyroscopes. The *B* value is two orders of magnitude smaller for the FOG than for a medium-cost MEMS gyroscopes and $t_{B}$ one order of magnitude greater for the FOG than for the medium-cost MEMS gyroscopes [23]. Consequently, the FOG biases are considerably more stable than the medium-cost MEMS gyroscopes biases.

In order to evaluate the magnitude of the FOG biases and their effect on the orientation estimation, the integration of 1 h of turn rate FOG measurements after correcting the Earth rotation has been computed. The FOG does not measure the transport rate since the aforementioned measurements were static, however it measures the rotation of the Earth, $\Omega_{e}$, which has a value of 7.29 × 10^−5^ rad s^−1^, approximately 15 ° h^−1^. Before integrating the turn rate measurements to compute the orientation, the Earth rotation has to be compensated as follows:

(26) $$\omega_{\mathrm{nb}}^{b}=\omega_{\mathrm{ib}}^{b}-C_{n}^{b}\;\cdot \;\omega_{\mathrm{in}}^{n},$$

being $C_{n}^{b}$ the transformation matrix from the navigation frame to the body frame and

(27) $$\omega_{\mathrm{in}}^{n}=\Omega_{e}\;\cdot \;{\left[0,\cos\left(\lambda \right),\sin\left(\lambda \right)\right]}^{T},$$

where λ is the latitude.

The resulting orientation angles, calculated without subtracting the biases estimation or applying updates, contain slight errors that do not exceed 1 ${}^{\circ }$ over 1 h in any angle. This error is due to the white noise and biases of the FOG which have not been compensated. A MEMS gyroscopes biases value of 0.1 ° s^−1^ yields to 360 ${}^{\circ }$ over 1 h. Since the FOG biases are orders of magnitude smaller and more stable than the medium-cost MEMS gyroscopes biases and the experiments last less than 1 h, we consider the error due to FOG biases negligible and the FOG turn rate measurements as quasi-error-free.

## 3. Results

In this section we will summarize the results of the experiments that aim to evaluate the effectiveness of using magnetic field measurements to estimate the biases of medium-cost MEMS gyroscopes. In order to properly observe the estimation process of the biases, we use quasi-error-free turn rate measurements and add a known constant bias value. This is detailed in the first part of the experiments, where we use the IMU DSP-1750 (KVH Technologies, Aiken, SC, USA).

The results of the first part of the experiments will be endorsed using measurements recorded with the MEMS MTw sensor. On the one hand, for the second part of the experiments the biases inherent to MEMS sensors are unknown. On the other hand, the results using real biases should be consistent to the first experiments where we use a constant bias model.

### 3.1. Evaluation with IMU DSP-1750 Measurements

**Figure 6:**
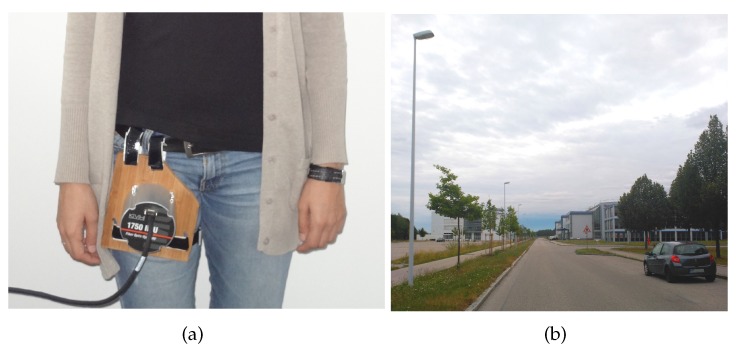
(**a**) The inertial data for the experiments analyzed in this section has been recorded with the IMU DSP-1750 attached to a wooden surface which is fastened to the leg, as if the sensor were introduced in the pocket. (**b**) Scenario of the experiments detailed in this section.

The IMU DSP-1750 is attached to the upper front part of the leg (see Figure 6a) as if it were introduced in the pocket.

As indicated in Section 2.2, we have added white noise v and biases b to the quasi-error-free turn rate measurements and a noise term $v_{\mu }$ has been added to the emulated magnetic measurements. These values, based on the MEMS gyroscopes and the magnetometer of the medium-cost sensor MTw from Xsens, are summarized in Table 2.

**Figure 7:**
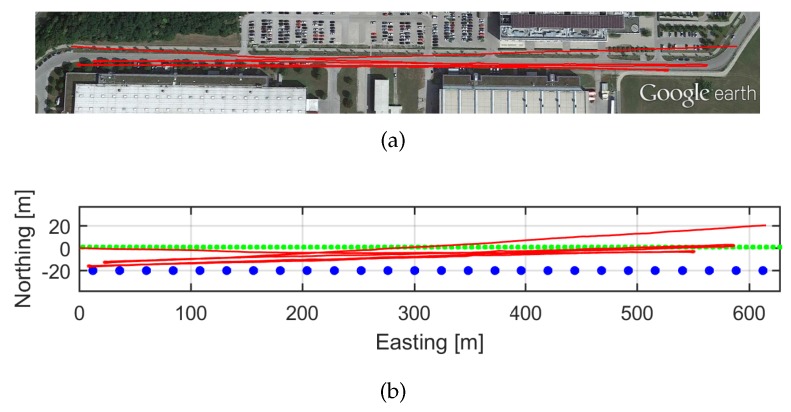
(**a**) Satellite image of the real walk scenario of the experiments. The red line represents the 39 min estimated trajectory over 3 km. (**b**) This figure shows in red the estimated trajectory, the blue points represent the parked cars and the green points represent the streetlights.

Figure 7a shows the satellite image overlay and the estimation of the trajectory depicted in red. The scenario of the walk is shown in Figure 6b. The walk duration is 39 min and it consists of a three round trip trajectory of 600 m on each direction, thus 3 km in total approximately. Figure 7b shows the estimated trajectory in red and the infinite cylinders representing the real streetlights and cars shown Figure 6b. The green points represent the streetlights and the blue points represent the parked cars from the template shown in Figure 4, that is replicated in the Easting direction to cover the complete trajectory. The post-processing described in Section 2.2 allows using the same recorded acceleration and turn rate measurements to emulate different magnetic field distributions at different locations on the Earth. The streetlights and cars will be used to emulate the perturbed magnetic field $H_{\pi }$, and disregarded to emulate homogeneous magnetic fields $H_{\infty }$. Thanks to this procedure we can evaluate the effect of the magnetic field distribution on the biases estimation, because the magnetic field is the only variable among all the experiments. The orientation estimation filter settings are also constant, therefore the *Differential Magnetic Field Update* has been applied with the same measurement noise values for homogeneous and perturbed magnetic measurements.

To obtain the trajectory of Figure 7, the orientation has been computed integrating the FOG turn rate measurements without subtracting the biases estimation or applying updates. Therefore, the FOG white noise and biases cause an error in the orientation, as previously explained. The Earth rotation has been compensated. Regarding the position computation, the algorithms described in [22] have been used for the step detection and step length computation.

In the following, the biases estimation with different magnetic field distributions will be analyzed as well as the effect of the biases estimation on the yaw angle. The first experiment consists of the biases estimation without using magnetic measurements. For the second experiment the *Differential Magnetic Field Update* is used under homogeneous magnetic fields. The rest of conditions keep the same as the first experiment. The third experiment replaces the homogeneous magnetic fields of the second experiment by a perturbed magnetic field. Last, the effect of using different magnetic field distributions on the yaw angle estimation is analyzed. The *Absolute Gravity Update* will be applied in all experiments of this work.

#### 3.1.1. Biases Estimation without Using Magnetic Field Measurements

This experiment aims at evaluating biases estimation process without using magnetic measurements. It is expected that the *Absolute Gravity Update* modifies directly the estimations of the roll and pitch angles, Equations (12) and (13). The yaw angle, however, is not observable through this update because it defines the rotation around the Up-axis, which is parallel to the gravity field (see Figure 2). The biases of the gyroscopes are also modified because they are used for the angle computation (Equations (4)–(6)).

**Figure 8:**
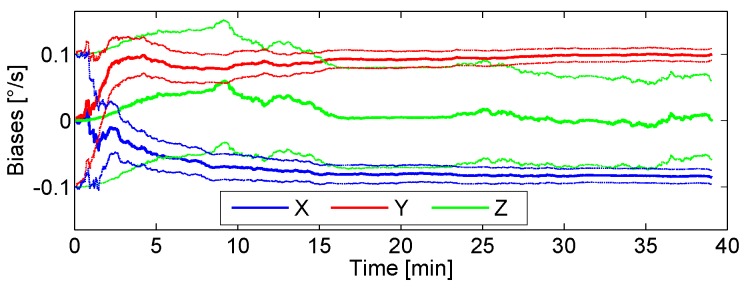
Biases estimation process using the *Absolute Gravity Update*. The blue line represents the biases estimation of the *x*-axis and the red and green the biases estimation of the *y*- and *z*-axis respectively. The thin lines represent the uncertainty.

Figure 8 shows the estimation of the biases for the three axes gyroscopes using only the *Absolute Gravity Update*. The figure shows that the bias of the *y*-axis is correctly estimated because the estimation converges to the set value summarized in Table 2. Even though the *x*-axis bias is estimated in the correct direction, the final value does not reach −0.1 ° s^−1^ in 39 min. A possible cause is the fact that the *Absolute Gravity Update* is only applied during the periods of zero or quasi-zero acceleration. These periods are longer or shorter depending on the walking speed of the pedestrian, tending to disappear if the pedestrian runs. For this experiment, the pedestrian travelled 3 km in 39 min. A walking speed of 4.6 km h^−1^ is considered medium-high. Additionally, it has to be considered that the acceleration measurements contain biases that have neither been estimated nor compensated. The *Absolute Gravity Update* alone is not able to aid the *z*-bias estimation because the periods of zero acceleration while the pedestrian is walking coincide with the leg of the pedestrian completely vertical. Therefore, the sensor *z*-axis within that periods is always aligned with the Up-axis when the update is active. The *Absolute Gravity Update* leads to a reduction of the uncertainty of the biases of the *x*- and *y*-axis, as the thin blue and red lines respectively show. As a consequence of the erroneous estimation of the bias in the *z*-axis, the yaw angle accumulates more error than roll and pitch. Therefore, further updates are needed in order to correctly estimate the *z*-axis bias.

#### 3.1.2. Biases Estimation Using Magnetic Measurements from Homogeneous Field

For this experiment the *Differential Magnetic Field Update* will be applied. This experiment aims at evaluating the influence of the magnetic field distribution for homogeneous fields in the biases estimation process. To that end, we have emulated different locations on the Earth, i.e., North Pole, Equator and Munich (Germany), for the same real walk described in this section.

**Figure 9:**
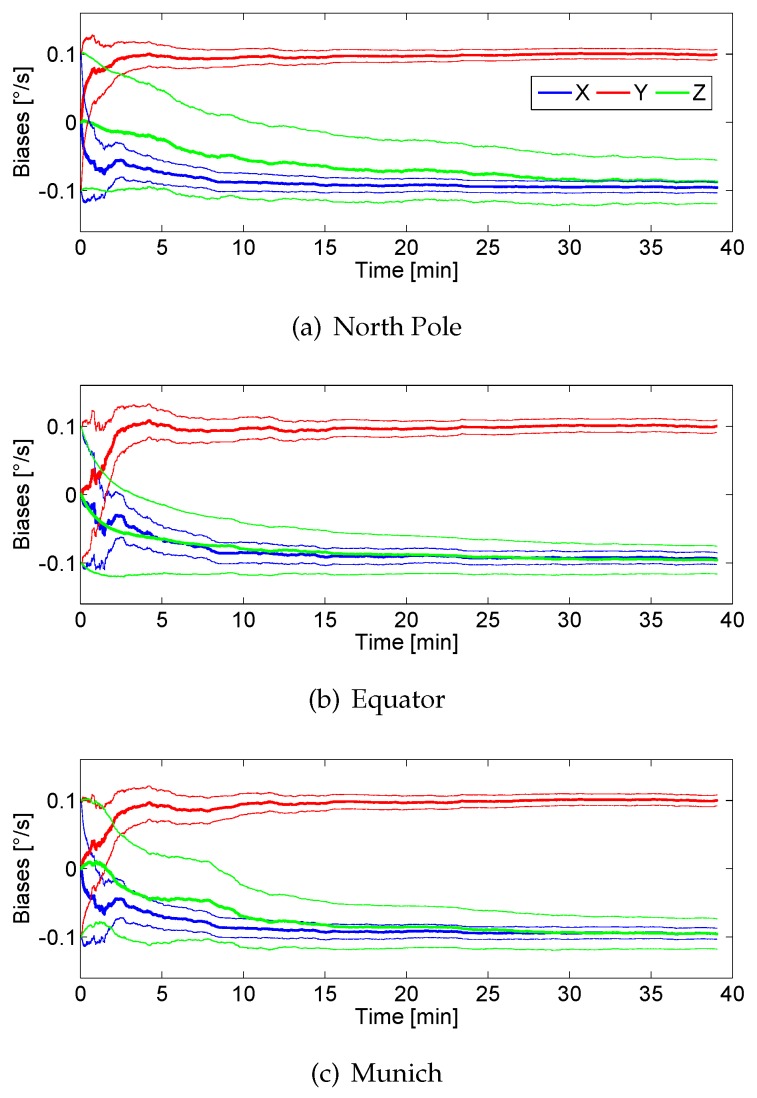
Biases estimation process using the *Absolute Gravity Update* and *Differential Magnetic Field Update* and considering $H_{\infty }$ of (**a**) North Pole (**b**) Equator and (**c**) Munich. The blue curve represents the biases estimation of the *x*-axis and the red and green the biases estimation of the *y*- and *z*-axis respectively. The thin curves represent the uncertainty.

As Figure 9 shows, in all cases the biases tend to the correct value specified in Table 2, however, slightly differences can be observed depending on the location of the pedestrian on the Earth. Figure 9a represents the biases estimation if the real walk represented in Figure 7 took place at the North Pole. The magnetic field is distributed only in the Up-axis, which is ideal to observe rotations around the *x*- and *y*-axis, however it is rotation invariant for the *z*-axis. The blue and red curves show a faster estimation process compared with the estimation that uses only the *Absolute Gravity Update*. Therefore, the *Differential Magnetic Field Update* also benefits the *x*- and *y*-biases estimation.

Theoretically, the distribution of the North Pole magnetic field does not improve the estimation of the *z*-axis bias, because rotations around the axis parallel to the Up-axis are not observable. However, as Figure 9a shows, the bias of the *z*-axis tends to the correct value, compared with the case where no magnetic measurements are used. The observation of the *z*-axis bias is possible thanks to the cyclic movement of the leg of the walking pedestrian, which makes the *z*-axis of the sensor not being constantly aligned with the Up-axis. Even if the estimation of the *z*-axis bias takes more time to converge than the other axes, these intermittent periods of no alignment caused by the movement of the leg favour the z-bias observation. In this case, it takes 29 min to reach the value −0.08 ° s^−1^.

The distribution of the magnetic field of the Equator does not allow observing rotations around the North-axis. However, the *y*-axis bias estimation is solved using only the *Absolute Gravity Update*. The magnetic field of the Equator allows observing rotations around the *x*- and *z*-axis. The result of Figure 9b yields that the bias of the *z*-axis has more chances to be observed than in the North Pole. In fact, it takes 10 min to reach the value −0.08 ° s^−1^. Figure 9c shows the biases estimation for the magnetic field distribution of Munich, which has an inclination angle of 64 ${}^{\circ }$ resulting in North and Up magnetic field components. As it can be observed, the estimation process of the biases is a mixture of North Pole and Equator. It takes 15 min for the z-bias estimation to reach the value −0.08 ° s^−1^.

In order to evaluate the influence of the direction of movement for a predetermined magnetic field distribution the orientation of the real walk has been modified to North-South, instead of the one shown in Figure 7a, which is East-West oriented. A slightly faster convergence of the *y*-axis bias can be observed if the trajectory is North-South oriented. This can be explained because rotations around the *y*-axis are easily observable when walking transversally to the magnetic field rather than walking in parallel. Changes in *x*- and *z*-bias estimations are not significant.

#### 3.1.3. Biases Estimation Using Magnetic Measurements from Perturbed Field

For this experiment, the *Differential Magnetic Field Update* will be applied. This experiment aims at evaluating the influence of magnetic perturbations on the biases estimation process. The homogeneous magnetic field, $H_{\infty }$, of the Equator has been perturbed by series of ferromagnetic objects spread along the trajectory, as Figure 7b shows.

**Figure 10:**
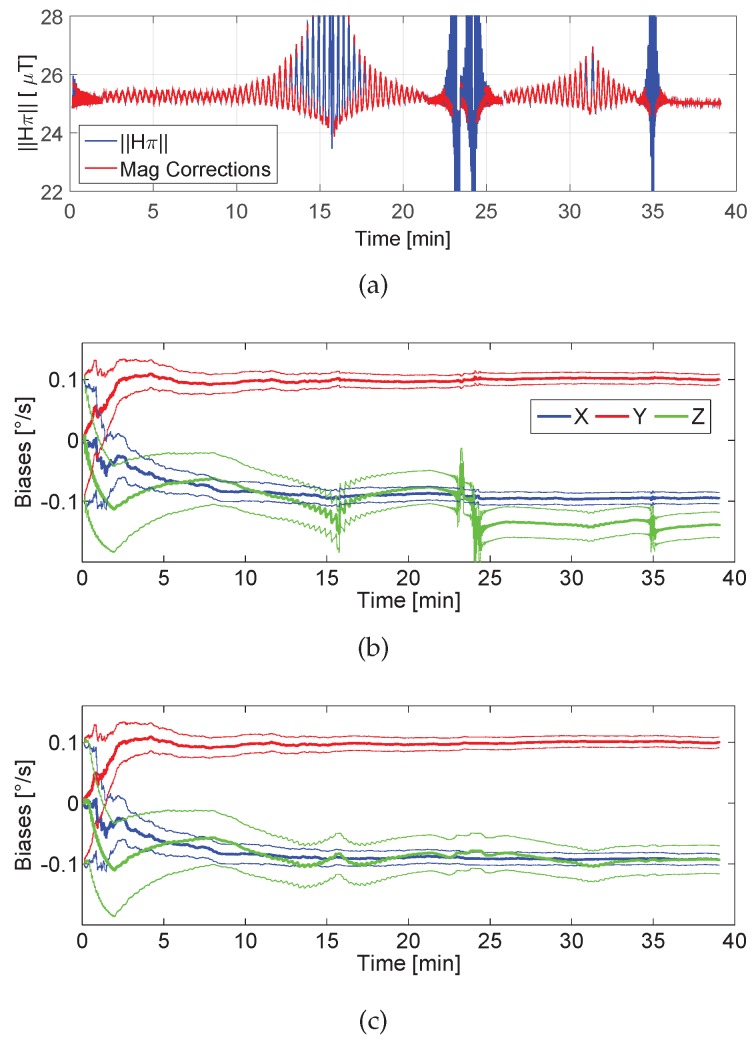
Biases estimation process considering $H_{\pi }$. (**a**) shows in blue the norm of the magnetic field and in red the periods where the *Differential Magnetic Field Update* is applied, (**b**) depicts the biases estimation using the *Absolute Gravity Update* and *Differential Magnetic Field Update* continuously and (**c**) shows the biases estimation using the *Absolute Gravity Update* and *Differential Magnetic Field Update* within the red highlighted periods.

Figure 10a shows the norm of the magnetic field measured over time during the experiment. If the magnetic field were homogeneous, its norm would be constant and equal to the field strength at the Equator, 25 μT. The perturbations seen in Figure 10a are due to streetlights at 24 min and 35 min, and cars at 16 min and 32 min (see Figure 7b). The magnitude of the perturbations depends on the proximity of the pedestrian to the ferromagnetic object.

The use of the *Differential Magnetic Field Update* is beneficial to estimate the *z*-axis bias in perturbed magnetic fields, since the estimation tends to the correct value specified in Table 2. However, if severe perturbed magnetic measurements are used, the *z*-axis bias estimation varies strongly to adapt the orientation estimation to the magnetic field distribution, as shown at 16 min, 24 min and 35 min, affecting also the *x*- and *y*-axis biases estimation. Figure 10c shows the biases estimation by using the *Differential Magnetic Field Update* only when the *magnetic disturbances detector* (see [26]) does not deactivate it. The red curve of Figure 10a shows the periods where the update is active. This analysis yields that it is convenient to use a *magnetic disturbances detector*, that results in a smoother *z*-axis bias estimation.

All in all, the *Differential Magnetic Field Update* is beneficial, since it causes the z-bias estimation to approach to the correct value of −0.1 ° s^−1^. However, it is convenient to implement a *magnetic disturbances detector* that strongly rejects perturbations, even though this implies that the *z*-axis bias spends more time to approach the bias value. For the case represented in Figure 10c, the *z*-bias needed 32 min to approach the correct value.

#### 3.1.4. Effect of the Biases Estimation on the Yaw Angle

**Figure 11:**
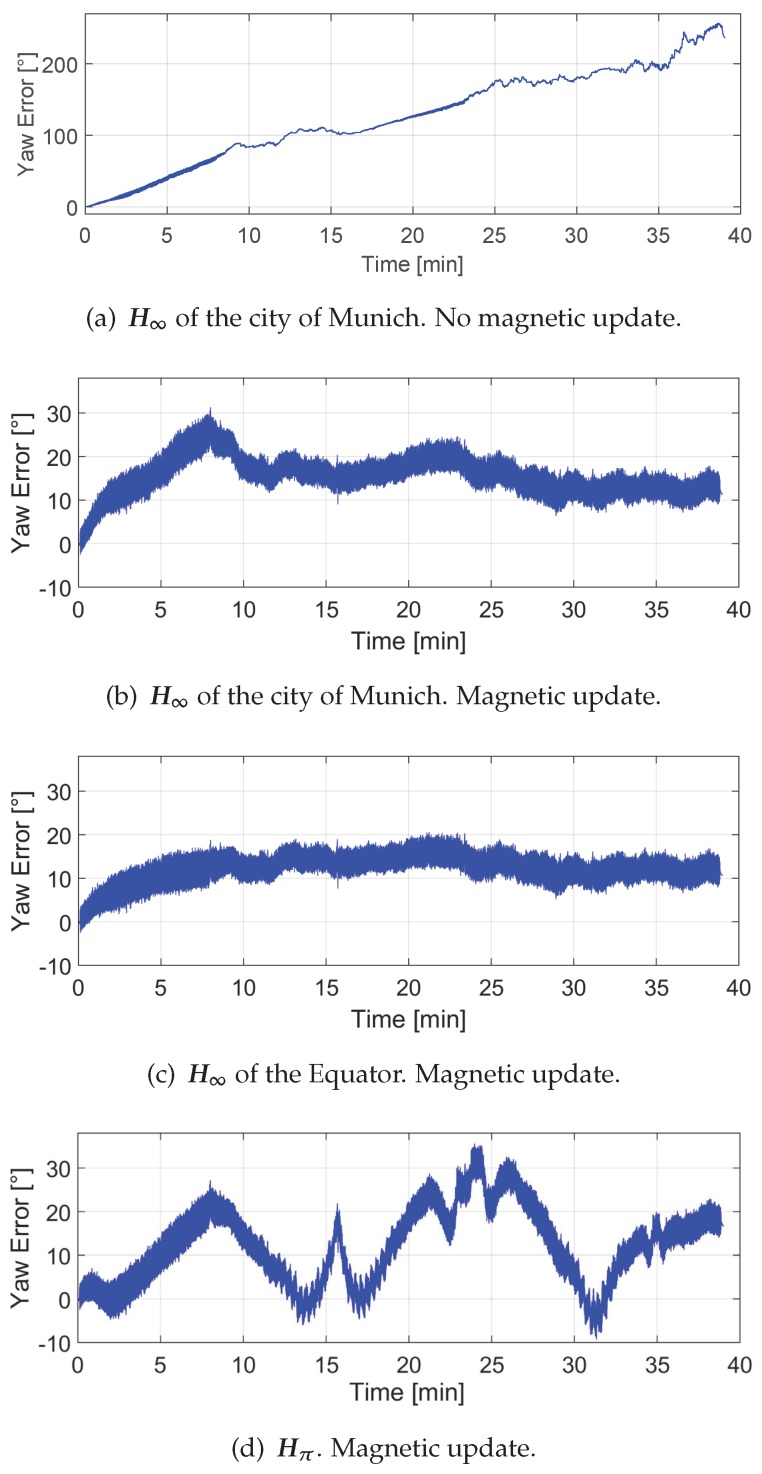
Error in the yaw angle estimation for the different magnetic scenarios with and without using the magnetic field measurements.Effect of the *z*-axis bias on the yaw angle

The *Absolute Gravity Update* is able to limit the error growth in roll and pitch angles; however, it has no positive effect on the yaw angle. Indeed, roll and pitch errors are stable after a long operation time, such as 39 min and the error of the yaw angle is constantly increasing as shown in Figure 11a. From the previous experiments we have learnt that the *Differential Magnetic Field Update* is capable of estimating the *z*-axis bias of the gyroscopes under different magnetic field distributions. The effect of the *z*-axis bias estimation on the yaw angle is evaluated in the following. The correct estimation of the *z*-bias should decrease the growing yaw angle error.

Figure 11b shows the yaw angle error under the homogeneous magnetic field corresponding to the city of Munich. As Figure 9c shows, the *z*-axis bias estimation is not stable within the first 15 min, therefore the yaw angle error has the same behaviour. It is remarkable that the *z*-axis bias estimation (see Figure 9c) is not smoothly approaching the value −0.1 ° s^−1^, thus the yaw angle error in Figure 11b is rapidly increasing and decreasing at the beginning.

Figure 11c shows the yaw angle error under the homogeneous magnetic field of the Equator. As clearly seen in Figure 9b, the *z*-axis bias estimation is rapidly approaching the correct bias value −0.1 ° s^−1^. Therefore, as expected, the yaw error does not ever grow and is stable after 10 min. The error after 10 min is not equal to 0 ${}^{\circ }$, because the z-bias estimation does not reach the correct bias value −0.1 ° s^−1^ and additionally the biases of the FOG have not been compensated.

Finally, Figure 11d shows the yaw angle error for a perturbed magnetic field. The *Differential Magnetic Field Update* has been activated by the *magnetic disturbances detector*. This case is the most similar to the real world, because it is not common to find homogeneous magnetic fields for indoors and urban scenarios. It has been shown that the effect of the perturbations caused by ferromagnetic objects is visible in the biases estimation (see Figure 10c) and therefore also in the yaw angle, as this figure shows. The oscillations in the z-bias estimation due to the perturbations are visible in the yaw angle estimation.

### 3.2. Evaluation with Medium-Cost MEMS Measurements

For this section, we recorded the measurements with the MEMS inertial sensors and magnetometer MTw. The aim of this section is endorsing the previous results obtained with emulated magnetic field measurements and quasi-error-free turn rate measurements with added constant biases. Thus, these experiments will include both bias components, stochastic and deterministic. The magnetometer embedded in the MTw sensor was calibrated before recording the new experiment. The calibration process took place in a disturbances-free environment: the sensor was manually moved describing random paths covering all directions. The recorded measurements form a shifted ellipsoid if the data is not calibrated. A least-squares algorithm is used to find the rotation, translation and scaling factor to bring the ellipsoid to a sphere with radius equal to 1 centered in the origin. The center of the sphere represents the biases of the three-axis magnetometer. The radius of the sphere is used to normalize the magnetic measurements to the local magnetic field intensity.

**Figure 12:**
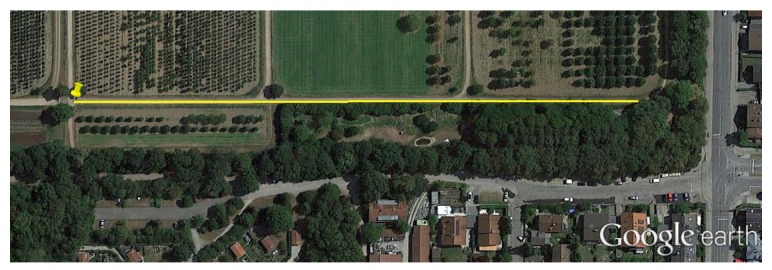
Disturbances-free scenario where the experiment of this section has been recorded. The initial and final point is highlighted with the yellow pin and the round trip trajectory is marked in yellow. The total length of the walk is 4.3 km over an elapsed time of 44 min.

The biases of the gyroscopes have been measured before and after finishing the walk. The walk has been recorded in the city of Munich in a disturbances-free scenario. The trajectory of the walk is highlighted in yellow in Figure 12 and consists of a round trip trajectory of 360 m section length repeated 12 times resulting in a 4.3 km trajectory covered in approximately 44 min. The yellow pin represents the initial and final point of the walk.

#### 3.2.1. Biases Estimation Using Magnetic Measurements from Real Homogeneous Field

The aim of this experiment is to use real magnetic field measurements with the *Differential Magnetic Field Update* and to estimate real biases. In order to obtain a reference value for the biases of the gyroscopes, the MTw sensor has been placed on the floor between 1.5 min and 2 min at the beginning and at the end of the walk. The averaged values of the initial and final static turn rate measurements are assumed to be the biases of the gyroscopes, because no other rotations are undergoing. For these measurements the influence of the Earth turn rate is disregarded due to its small value and the biases are considered constant because the bias stability of the MEMS MTw gyroscopes, *B*, is much greater than 2 min. The biases evolve during the walk, thus the initial and final value does not have to be the same. Table 3 shows the observed biases.

**Figure 13:**
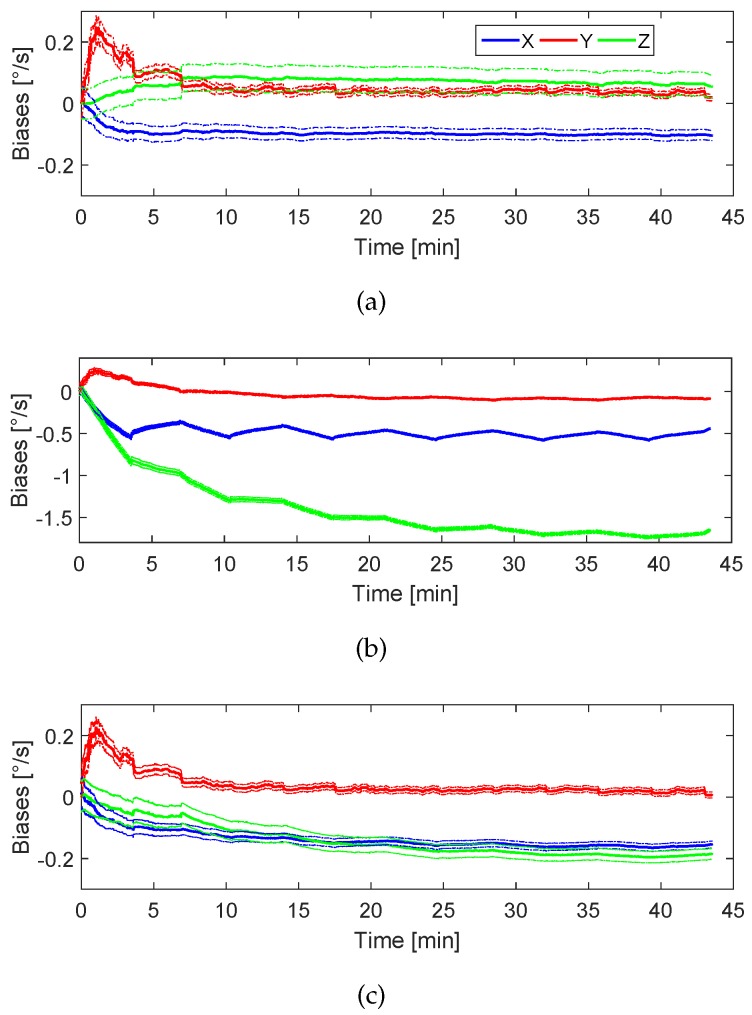
Biases estimation over time considering the real magnetic field of Munich. (**a**) only applying the *Absolute Gravity Update*. (**b**) applying both updates with uncalibrated magnetic measurements and (**c**) applying both updates with calibrated magnetic measurements.

Figure 13a shows the biases estimation process using only the *Absolute Gravity Update*. The biases corresponding to the *x*- and *y*-axis are correctly estimated, because their values match the previously measured biases summarized in Table 3. The *z*-axis bias estimation is influenced by the *Absolute Gravity Update*, however, it does not converge to the correct value. Its uncertainty, represented with the thin green lines, does not decrease. This result fully corresponds to the analysis of the previous experiments when only applying the *Absolute Gravity Update*.

Figure 13b shows the biases estimation process applying the *Absolute Gravity Update* and the *Differential Magnetic Field Update* continuously by using the uncalibrated raw magnetic measurements. The *Differential Magnetic Field Update* has been used continuously because the update is always active under homogeneous magnetic fields. The estimated *z*-axis bias reaches a value of almost −1.7 ° s^−1^, being the measured value −0.22 ° s^−1^ at the end of the walk (see Table 3). The *x*-axis bias is also negatively affected by the magnetic field measurements, causing an oscillation around −0.5 ° s^−1^ when it should be almost constant at −0.12 ° s^−1^. This result shows the importance of calibrating the magnetometer sensor before applying any magnetic correction even under homogeneous magnetic fields.

Figure 13c shows the biases estimation process applying the *Absolute Gravity Update* and the *Differential Magnetic Field Update* continuously, because the measurements have been recorded under a homogeneous magnetic field. For this experiment, the magnetometer measurements have been calibrated. The figure shows how the bias corresponding to the *z*-axis slowly tends to the measured value (see Table 3) without damaging the *x*- and *y*-axis biases estimation. This result endorses the previous analysis carried out with emulated magnetic field measurements corresponding to different locations on the Earth. However, it is important to note that even under homogeneous magnetic fields, that implies applying the *Differential Magnetic Field Update* continuously, the measured z-bias value in Figure 13c has not been reached after 44 min. Therefore, the time needed for the *Differential Magnetic Field Update* to yield a correct estimation of the z-bias is high and it increases under non-homogeneous magnetic fields, which are common indoors.

#### 3.2.2. Effect of the Biases Estimation on the Trajectory

**Figure 14:**
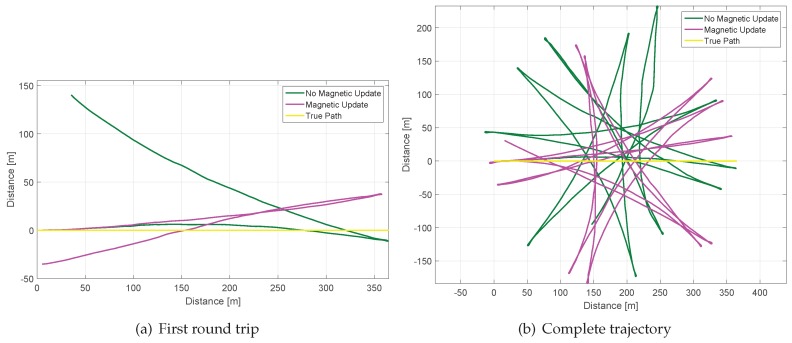
The error-free trajectory of the experiment of this section is shown in yellow. The magenta curve corresponds to the trajectory by applying both updates and the green curve is the result of applying only the *Absolute Gravity Update*.

The consequence of not estimating correctly the z-bias or the consequence of a very slow estimation is an accumulated error on the yaw angle, as previously shown. The error in the yaw angle leads to distorted trajectories, as Figure 14 shows.

Figure 14 shows in magenta the resulting trajectory by applying the *Absolute Gravity Update* and the *Differential Magnetic Field Update* and in green result of applying the *Absolute Gravity Update* and no magnetic corrections. The error-free trajectory corresponding to the experiment of this section is highlighted in yellow. Figure 14a shows the first round trip while Figure 14b shows the complete trajectory. Figure 14a shows in magenta the trajectory which has accumulated an error in the yaw angle of approximately 20 ${}^{\circ }$ after the first round trip. In green it is shown the first round trip trajectory with an accumulated error in the yaw angle of approximately 50 ${}^{\circ }$. For this trajectory, this traduces to 30 m and 140 m of displacement, respectively (measuring the error in position as a straight line between initial and final point). Figure 14b shows the complete trajectory, that has accumulated an error in the yaw angle of approximately 160 ${}^{\circ }$ for the magenta curve and 270 ${}^{\circ }$ for the green curve.

Therefore, the trajectory estimation also shows that the *Differential Magnetic Field Update* has a positive effect by fostering the estimation of the biases of the gyroscopes, especially the *z*-axis bias. However, a great error is still accumulated during the long time needed for this update to obtain a correct estimation.

## 4. Conclusions

This work aims at evaluating experimentally the effectiveness of using magnetic field measurements to estimate the biases of medium-cost MEMS gyroscopes. The use of magnetic field measurements for estimating the gyroscopes biases has been proposed in the state of the art, but not proved. We focus on pedestrian navigation and we place the sensor in the front pocket of the trousers. First, error-free turn rate measurements with known added biases and emulated magnetic field measurements have been used. Last, these results have been supported by measurements recorded with medium-cost MEMS sensors.

We prove that the biases estimation without using magnetic field measurements is possible for the *x*- and *y*-axis. However, the z-bias remains unobservable only correcting with the gravity acceleration. Different locations on the Earth, i.e., North Pole, Equator and Munich, are evaluated with homogeneous magnetic fields. We conclude that thanks to the movement of the leg of the pedestrian while walking, it is possible to correctly estimate the z-bias even in the less favorable location on Earth. However, the magnetic field distribution influences the time needed to reach the correct value of the biases. We demonstrate that it is possible to obtain a good estimation of the biases under magnetically perturbed environments. However, it is convenient to use a disturbances detector that selects constant or quasi-constant magnetic field periods rather than using perturbed magnetic measurements continuously. The effect of the estimation of the biases on the yaw angle is analyzed under different magnetic scenarios, showing that correct estimations of the biases favour successful orientation estimations. Additionally, measurements recorded with a medium-cost MEMS are analyzed. The obtained results match the previous experiments carried out with emulated magnetic field measurements and known constant bias. Lastly, the effect of the estimation of the biases is evaluated also using the computed trajectory, where lower errors are shown by using magnetic field measurements.

All in all we conclude that the correction based on magnetic field measurements proposed in the state of the art has a positive effect on the biases estimation. However, it has a high limited action by lowering the yaw angle error and the error on the computed trajectory for medium-cost MEMS sensors even under homogeneous magnetic fields. The effect is more reduced on magnetically perturbed scenarios, e.g., indoors. 

## Figures and Tables

**Table 1:** Homogeneous Magnetic Fields, $H_{\infty }$, measured in μT.

North Pole	Equator	Munich
65 · [0, 0, −1]${}^{T}$	25 · [0, 1, 0]${}^{T}$	48 · [0, cos(64 ${}^{\circ }$), –sin(64 ${}^{\circ }$)]${}^{T}$

**Table 2:** Values of Noise and Biases.

	*v* [${}^{\circ }$ s${}^{-1}$]	*b* [${}^{\circ }$ s${}^{-1}$]	$v_{\mu }$ [μT]
*x*-axis	0.1	−0.1	0.015
*y*-axis	0.1	0.1	0.015
*z*-axis	0.1	−0.1	0.015

**Table 3:** Computed Biases, measured in ° s^−1^, for the MTw MEMS Gyroscopes.

	*x*-axis	*y*-axis	*z*-axis
Start	−0.12	0.06	−0.12
End	−0.13	0.05	−0.22
